# Sequencing and analysis of the complete mitochondrial genome of *Mansonia uniformis* (Dipera: Culicidae)

**DOI:** 10.1080/23802359.2019.1704638

**Published:** 2020-01-10

**Authors:** Junhua Tian, Bin Yu, Xianfeng Shi, Huan Liang, Dehuan Wang, Mihong Ge

**Affiliations:** a Wuhan Centers for Disease Control and Prevention, Wuhan, PR China;; b Institute of Crop of Wuhan Academy of Agricultural Sciences, Wuhan, PR China

**Keywords:** Mitochondrial genome, *Mansonia uniformis*, Culicidae, phylogeny

## Abstract

In this study, we sequenced and analyzed the complete mitochondrial genome of *Mansonia uniformis*, and this is the first report on the genus Mansonia. The circular mitogenome is 15,603 bp long and contains 13 protein-coding genes (PCGs), 22 tRNA genes, 2 ribosomal RNA genes, and a A + T-rich control region. Most PCGs start with ATN codons, and end with TAA, except for *COX1* starting with TCG codons and *COX2* ending with a single thymine stop codon. The phylogenetic tree based on the *COX1* gene showed that *M. uniformis* formed a monophyletic clade, sister to other seven genus from the subfamily Culicinae.

Culicidae, known as mosquito, constitutes 41 genera, 201 subgenera, and 3573 species in the world. A total of 419 species have been recorded from China, representing 20 genera and 63 subgenus (Fu and Chen 2018). The subfamily Culicinae is considered to be the largest and polyphyletic with a total of 3048 species belonging to 38 genera and 11 tribes (Harbach 2007). However, the current knowledge about the taxonomy and systematics of Culicinae is still limited (Reidenbach et al. 2009). In this article, we first present the mitogenome sequence of the genus Mansonia, namely *Mansonia uniformis*, and hope this sequence could contribute to further studies on molecular phylogenetic analysis and accurate identification.

In this study, the samples were collected in Wuhan City, Hubei province, China (30°42′35″N, 114°28′11″E) and stored in Wuhan Academy of Agricultural Sciences in −80 °0 Ultra-low Temperature Freezer (Sample code is ZWSmosquito-20190522). High-throughput sequencing method (Tian et al. 2019) was employed to determine the complete mitogenome of *M. uniformis* (accession no. MN342085) with further bioinformatic analysis. The overall base composition (15,603 bp) is 39.26%, 38.17%, 14.15%, and 8.42% for A, T, C, and G, respectively, with the A + T content of 77.43%. It contains typical 13 protein-coding genes (PCGs), 2 rRNA genes (12S and 16S rRNA), 22 transfer RNA genes (tRNA), and a control region. Its gene organization, order, and orientation are similar to all other available mitogenomes in mosquito species (Behura et al. 2011; Hardy et al. 2016), except for *Dixella aestivalis* from the subfamily Dixinae (Briscoe et al. 2017).

Most PCGs start with typical ATN codons (seven ATG, three ATT, one ATC, and one ATA) and end with standard canonical TAA as their termination codons, except for *COX1* starting with TCG codons and *COX2* ending with a single thymine stop codon. The initiation codon TCG was commonly observed in other available Diptera mitochondrial genomes (Li et al. 2016; Ge et al. 2019). The length of the 22 tRNA genes varies from 64 to 72 bp. Eight tRNA genes (tRNA*^Gln^*, tRNA*^Cys^,* tRNA*^Tyr^,* tRNA*^Phe^*, tRNA*^His^*, tRNA*^Pro^*, tRNA*^Leu^*, and tRNA*^Val^*) were distributed on the light strand. Totally 17 locations of overlapping sequence are between neighboring genes, ranging from 1 to 8 bp in length, and 51 bp in total. Furthermore, the lengths of spacer sequences are 1–18 bp in 11 locations, and 62 bp in total. 12S and 16S rRNA genes are located between the tRNA*^Leu^* and the control region, and separated by the tRNA*^Val^* gene. The 764 bp control region is located between the 12s rRNA and tRNA*^Ile^*–tRNA*^Gln^*–tRNA*^Met^* cluster, with the base composition of 43.72% A, 46.99% T, 6.54% C, and 2.75% G.

The phylogenetic analysis was performed by MEGA version 6.06 program (Tamura et al. 2013) based on *COX1* gene sequences of *M. uniformis* with 16 closely related species, and *Dixella aestivalis* used as the outgroup (Figure 1). The phylogenetic tree showed that *M. uniformis* first clustered together with five species of genus Culex, and then they constituted a sister-group relationship with other four genus. The results supported that the genus Mansonia should be classified into the subfamily Culicinae.

**Figure 1.**
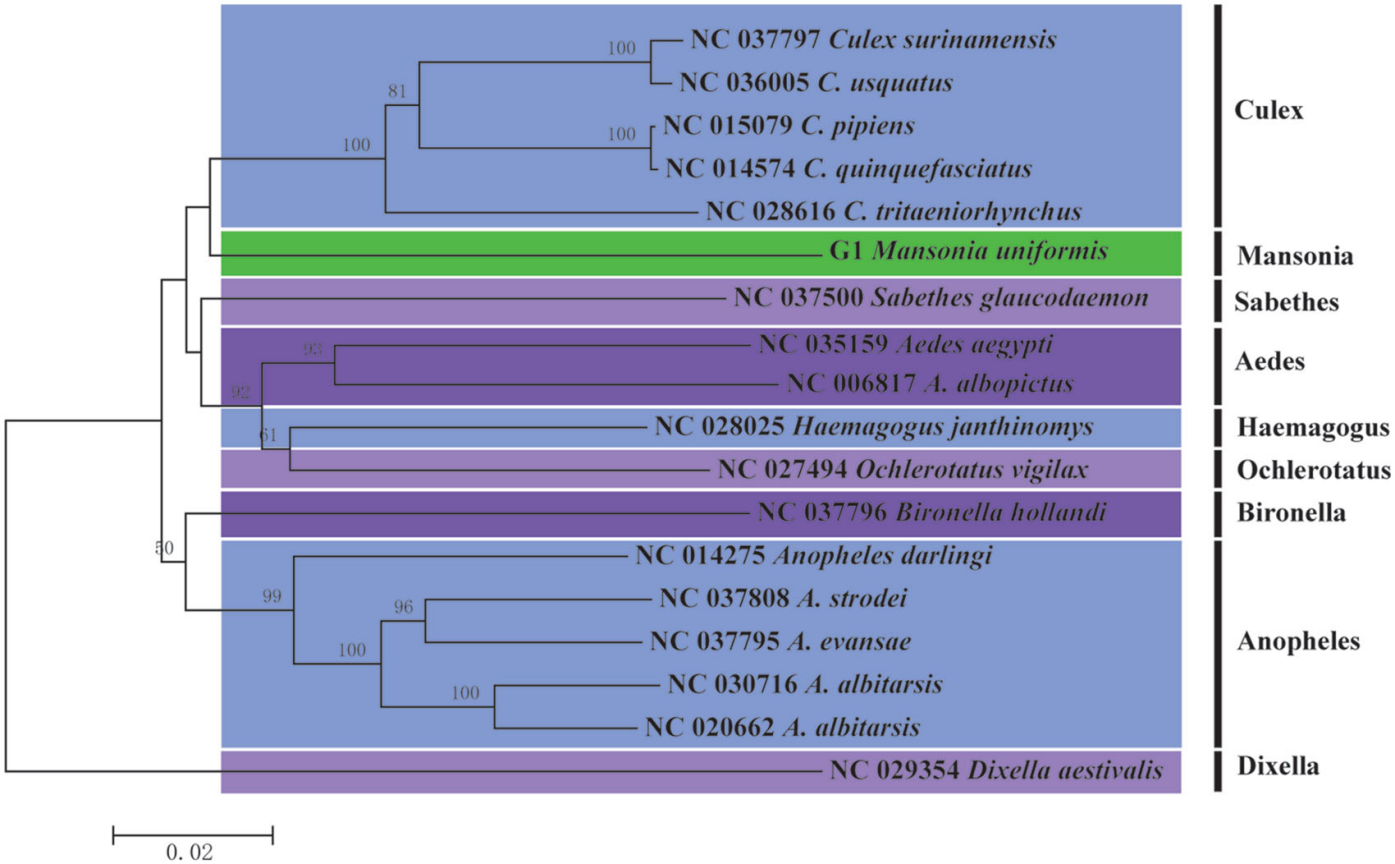
The neighbor-joining tree of *M. uniformis* based on *COX1* gene sequences with 16 closely related species of 7 genus *Culex*, *Sabethes*, *Aedes*, *Haemagogus*, *Ochlerotatus*, *Bironella*, and *Anopheles* from the subfamily Culicinae. Genus was *Dixella* used as outgroup. The tree was constructed by MEGA 6.06 with 1000 bootstraps.
